# Both Optimal Matching and Procedure Duration Influence Survival of Patients after Unrelated Donor Hematopoietic Stem Cell Transplantation

**DOI:** 10.1155/2012/873695

**Published:** 2012-10-22

**Authors:** Sylwia Mizia, Dorota Dera-Joachimiak, Malgorzata Polak, Katarzyna Koscinska, Mariola Sedzimirska, Andrzej Lange

**Affiliations:** ^1^Division of the National Bone Marrow Donor Registry, Lower Silesian Center for Cellular Transplantation, Grabiszynska 105, 53-439 Wroclaw, Poland; ^2^Institute of Immunology and Experimental Therapy, Polish Academy of Sciences, 53-114 Wroclaw, Poland

## Abstract

Eighty-six patients suffering from hematological malignancies, immunodeficiencies, and aplastic anemias received alloHSCT from unrelated donors. Donors were selected from the BMDW files and further matching was performed according to the confirmatory typing procedure with the use of PCR SSP and that based on sequencing. The time from the clinical request of the donor search to the final decision of clinicians accepting the donor was from 0.3 to 17.8 months (median 1.6). Matching was analyzed at the allele level, and 50, 27, and 9 donor-recipient pairs were 10/10 matched, mismatched in one or more alleles, respectively. In an univariate analysis we found better survival if patients were transplanted: (i) from donors matched 10/10 (*P* = 0.025), (ii) not from female donor to male recipient (*P* = 0.037), (iii) in female donation from those with ≤1 pregnancy than multiparous (*P* = 0.075). Notably, it became apparent that duration of the confirmatory typing process affected the survival (HR = 1.138, *P* = 0.013). In multivariate analysis only the level of matching and the duration of the matching procedure significantly affected the survival. In conclusion, the duration of the matching procedure in addition to the level of matching should be considered as an independent risk factor of survival.

## 1. Introduction

The number of allogeneic hematopoietic stem cell transplantations (alloHSCTs) from unrelated donors has increased over the years and in Europe reached 7098 in 2010 (EBMT Survey on Transplant Activity 2010). This was possible due to the improvement in international cooperation in donor-recipient matching procedures facilitated by the Bone Marrow Donors Worldwide (BMDW) files [1] and implementation of the European Marrow Donor Information System (EMDIS) in a number of countries. The priority of the search procedure is to identify the optimally matched donor for patients badly needing hematopoietic stem cell transplantation (HSCT). Quite recently the pace of the matching procedure has improved due to the use of computer-assisted communication systems including the EMDIS. However, still some time is needed, especially when the process of searching for a fully matched donor is prolonged. Previously published studies showed that the time needed to identify an acceptable donor is associated with a profile of HLA alleles being prolonged in cases with rare haplotypes [2–4]. Prolonged search may result in postponing transplantation in some cases that become medically unfit in the meantime. This may be due to various medical reasons including relapse and consequently, unless successfully treated, advancing in the stage of the disease. Tiercy et al. [4] showed that patients categorized in the group with a high probability of finding an optimal 10/10 matched donor have better survival than those with intermediate or low probability. Here, we study the impact of the actual length of the search procedure on the outcome of alloHSCT.

## 2. Materials and Methods 

### 2.1. Patients

In this study we analyze the outcome of 86 patients transplanted in our institution from unrelated donors in years 2004–2010. The patients suffered from hematological malignancies (80%), immunodeficiencies (15%), and aplastic anemias (5%). The group consisted of 39 (45%) females and 47 (55%) males aged from 0.6 to 59 years (median 28.5) and received marrow (6) or PBPC (80) from female (40) and male (46) donors (Table 1).

### 2.2. Histocompatibility Testing and Search Strategy

The donor-recipient matching procedure commissioned to the National Polish Bone Marrow Donor Registry (NPBMDR), a part of the Lower Silesian Center for Cellular Transplantation, was conducted according to two principles: (i) a donor should be compatible in human leukocyte antigen (HLA) with a patient at a high-resolution level of typing considering five loci (A, B, C, DR, and DQ) and (ii) among donors with similar HLA characteristics, residents of Poland, and if absent those from neighboring countries are chosen with priority [5, 6]. Donors were selected from the BMDW files with an HLA-compatible potential with a priority according to the distance principle policy. Further matching procedures were performed as follow: (1) registries having potential donors are conducted to confirm the donor availability and if so a blood sample is requested for confirmatory typing (CT), (2) as soon as blood is received high resolution typing of a potential donor is performed with the use of PCR SSP and that based on sequencing, and the same procedure is applied to the recipient, (3) the transplant center is asked for acceptance of a donor which may result in a request for further search, (4) the above procedures are performed in an iterative manner. 

The time from the beginning of the search process, the level of matching and the outcome of transplantation were recorded and statistically evaluated.

### 2.3. Data Collection

The outcome of transplantation was followed and registered in a database according to the EBMT Med-A form requirements. The overall survival of patients receiving alloHSCT from unrelated donor was evaluated using the already known factors including level of HLA matching, female-to-male donation, number of female donor pregnancies, age of donors and CMV serostatus and in addition the duration of the matching procedure.

### 2.4. Statistical Analysis

Statistical analysis was conducted using STATISTICA v.10. The associations between two variables were tested by Chi-square test, with Yates' correction if appropriate, for categorical variables and Mann-Whitney *U* test for categorical and continuous variables. The overall survival was analyzed by the Kaplan-Meier method, log-rank test, and parametric survival models [7, 8]. The likelihood of committing a type 1 error was set to 0.05. 

## 3. Results

All patients were typed at the level of a primary workup in a majority of cases. However, in 15% of cases patients were typed when it was clinically apparent that the transplant was badly needed. The time of the donor search varied from 0.3 to 17.8 months (median 1.6). Analysis of the level of matching at the point of clinical acceptance revealed that 50, 27, and 9 donor-recipient pairs were 10/10 matched, mismatched in one or more alleles, respectively.

The overall survival was significantly higher for patients transplanted from donors matched at the level of 10 specificities (2-year survival rates of matched and mismatched donors: 59% versus 38%, respectively; log-rank test *P* = 0.025) and transplanted other than from female donor to male recipient (2-year survival rates: 57% versus 32%, respectively; log-rank test *P* = 0.037). Survival curves of patients transplanted from female donors with no or 1 pregnancy tended to be higher than those reflecting the effect of donation from multiparous women (2-year survival rates: 53% versus 39%; log-rank test *P* = 0.075). 

Notably, it became apparent that duration of the searching process (mth) affected the survival (Cox model: hazard ratio HR = 1.138, *P* = 0.013). The results of univariate statistical analysis are shown in Tables 2 and 3.

**Figure 1:**
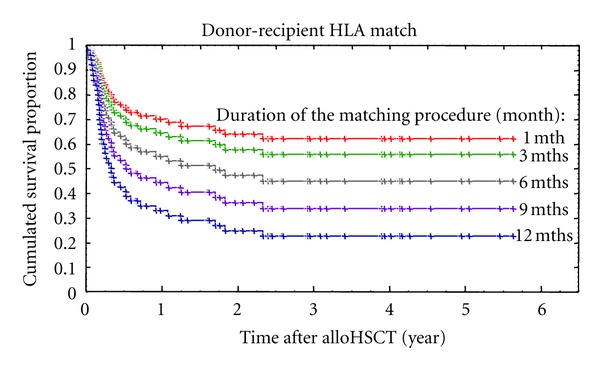
Survival curves of patients receiving alloHSCT from matched unrelated donor with respect to the duration of the matching procedure (as predicted according to the model).

**Figure 2:**
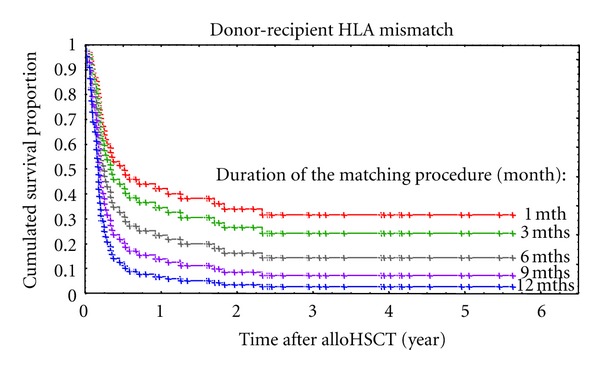
Survival curves of patients receiving alloHSCT from mismatched unrelated donor with respect to the duration of the matching procedure (as predicted according to the model).

In multivariate analysis only the level of matching and the duration of the matching procedure significantly affected the survival in an independent fashion (Cox model: HR = 2.422, *P* = 0.007 and HR = 1.109, *P* = 0.045, resp.) (Table 4). Multivariate analysis was used to calculate the coefficients reflecting the impact of different variables on the overall survival. More thorough analysis of the study group revealed that the duration of the searching process was significantly longer in patients having as compared to those lacking the presence of rare haplotypes and/or rare B-C or DR-DQ associations defined according to our published study (median: 3.1 versus 1.5 months, Mann-Whitney *U* test *P* = 0.001) [5]. Only 10% of patients with common HLA haplotypes waited longer than 3 months for a conclusion of the search process due to the prolonged donor activation time resulted, for example, from a withdrawal of a donor from the registry. In addition, we analyzed the presence of the progression in stage of the disease during the search process. It became apparent that proportions of patients who advance in stage of the disease were similar in patients with a short and a longer search process (median cut-point: 11% versus 14%, Chi-square test *P* = 0.865). This shows that in both groups there were patients with diseases at similar levels of relapse/progression potential. Time from the diagnosis to transplantation is influenced by several factors, including biology of underlying diseases and willingness of patients to undergo transplantation as an optional treatment. However, patients with a long time between the diagnosis and transplantation in the more homogeneous group of acute leukemias had more frequently rare alleles and/or B-C or DR-DQ associations than those being transplanted sooner after diagnosis (1-year cut-point: 50% versus 14%, Chi-square test *P* = 0.035). Therefore, length of the search process and the level of matching are major factors affecting post-HSCT survival. It enabled the development of a model predicting survival according to the level of matching and the time of the search process. Figures 1 and 2 show the predicted survival curves. 

**Figure 3:**
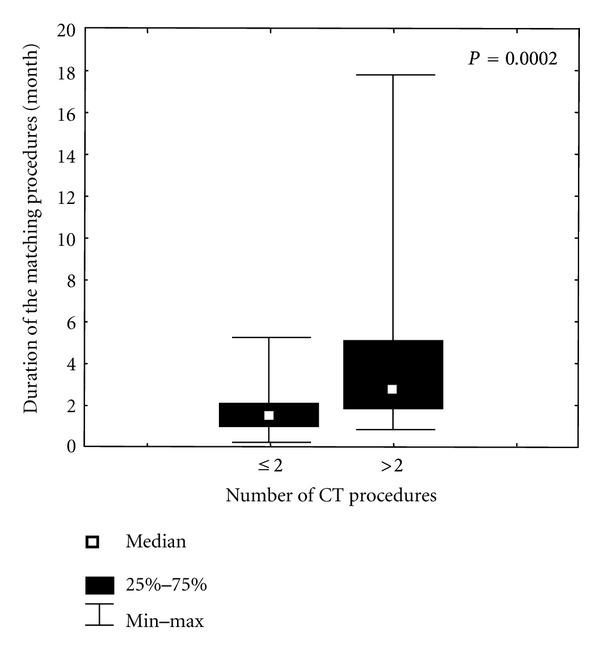
The duration of the matching procedure with respect to the number of CT procedures.

In addition we investigated whether time of the search procedure was affected by the number of matching attempts. It became apparent that more than two CT procedures resulted in a significant prolongation of the donor search completion (median: 1.5 versus 2.7 months, Mann-Whitney *U* test *P* = 0.0002; Figure 3). 

## 4. Conclusions

Data recorded in this study enabled us to confirm already known factors, namely, number of pregnancies and female-to-male donation, as those affecting survival after HSCT. This observation, concordant with other studies [9], shows that the donor-recipient pairs presented in this paper share similar characteristics with other reported HSCT groups of donors and recipients. Also it is apparent from the present study that the level of HLA matching plays an important role. This is also a well-known observation [10]. Keeping in mind the latter data, transplant centers frequently focus on the level of matching, neglecting the time needed for a prolonged procedure if the matching process is rather complex. Indeed, the time from the beginning until the completion of the search significantly depends on the number of confirmatory typing procedures performed. The novel aspect of the present paper is the finding that time needed for optimal match adversely affects the survival. Therefore, an optimal match reached after prolonged time results in a similar survival as that not optimal but completed promptly. Several previously published studies suggested ways to predict the length of the process on the basis of the HLA specificity profile in patients. This was also shown in the present group as patients with HLA rarities waited longer. Tiercy et al. [4] documented poorer survival in patients with rare alleles and B-C or DR-DQ associations. In the present study survival was analyzed not according to the HLA specificities associated with prediction but independently of any specific factors; just length of the search process was taken as a variable. Indeed, HLA rarities play an important role, but also other factors may be associated. Ten percent of patients with rather common HLA specificities waited for the search conclusion longer than 3 months. The reason of such delay is not entirely clear, but withdrawal of a potential donor from the registry may serve as an example.

The present study offers a rationale for the observation in the paper by Heemskerk et al. [11] that to achieve transplant results in the range of sibling transplantations the search procedure should be similarly time consuming. 

## Figures and Tables

**Table 1:** Patients' characteristics.

Recipient age (y), median (range)		28.5 (0.6–59)
Diagnosis, no. (%)	Hematological malignancy	73 (80)
	Immunodeficiency	9 (15)
	Aplastic anemia	4 (5)

Donor age (y), median (range)		34 (19–59)

Donor-recipient sex match, no. (%)	Female to male	23 (27)
	Other	63 (73)

Number of pregnancies in female donors, no. (%)	0-1	21 (54)
	>1	18 (46)

Donor-recipient CMV serostatus match, no. (%)	Positive-negative	10 (38)
	Negative-positive	26 (72)

Donor origin, no. (%)	Poland registry	14 (17)
	Europe foreign registry	63 (73)
	Other world registries	9 (10)

Donor-recipient HLA matching, no. (%)	Matched	50 (58)
	Mismatched	36 (42)

Number of CT procedures, no. (%)	≤2	58 (73)
	>2	22 (27)

Duration of the matching procedure (mth), median (range)		1.6 (0.27–17.8)

Hematopoietic stem cell source, no. (%)	Bone marrow	6 (7)
	Peripheral blood	80 (93)

**Table 2:** Univariate analysis (discrete variables).

		No.	Overall survival (2-yr survival, %)	*P*-value
Donor-recipient HLA matching	Matched	50	58.9	0.025
	Mismatched	36	37.7	

Donor-recipient sex match	F-M	23	31.8	0.037
	Other	63	56.6	

Number of pregnancies in female donors	0-1	21	52.5	0.075
	>1	18	38.9	

Donor-recipient CMV serostatus match	Positive-negative	10	58.3	0.479
	Negative-positive	26	48.8	

**Table 3:** Univariate analysis (continuous variables).

	HR	*P* value
Donor age (y)	1.004	0.775
Duration of the matching procedure (mth)	1.138	0.013

**Table 4:** Multivariate analysis.

	HR	*P* value
Donor-recipient HLA matching (mismatched)	2.422	0.007
Duration of the matching procedure (mth)	1.109	0.045
